# Family medicine training in Africa: Views of clinical trainers and trainees

**DOI:** 10.4102/phcfm.v10i1.1638

**Published:** 2018-04-12

**Authors:** Louis S. Jenkins, Klaus von Pressentin

**Affiliations:** 1 Department of Family and Emergency Medicine, Stellenbosch University, South Africa; 2 George Regional Hospital, Eden, Western Cape Department of Health, South Africa; 3 Mossel Bay Hospital, Eden, Western Cape Department of Health, South Africa

## Abstract

**Background:**

This article reports on the findings of a workshop held at the joint 5th World Organisation of Family Doctors (WONCA) Africa and 20th National Family Practitioners Conference in Tshwane, South Africa, in 2017. Postgraduate training for family medicine in Africa takes place in the clinical workspace at the bedside or next to the patient in the clinic, district hospital or regional hospital. Direct supervisor observation, exchange of reflection and feedback, and learning conversations between the supervisor and the registrar are central to learning and assessment processes.

**Objectives:**

The aim of the workshop was to understand how family medicine registrars (postgraduate trainees in family medicine) in Africa learn in the workplace.

**Methods:**

Thirty-five trainers and registrars from nine African countries, the United Kingdom, United States and Sweden participated. South Africa was represented by the universities of Cape Town, Limpopo, Pretoria, Sefako Makgatho, Stellenbosch, Walter Sisulu and Witwatersrand.

**Results:**

Six major themes were identified: (1) context is critical, (2) learning style of the registrar and (teaching style) of the supervisor, (3) learning portfolio is utilised, (4) interactions between registrar and supervisor, (5) giving and receiving feedback and (6) the competence of the supervisor.

**Conclusion:**

The training of family physicians across Africa shares many common themes. However, there are also big differences among the various countries and even programmes within countries. The way forward would include exploring the local contextual enablers that influence the learning conversations between trainees and their supervisors. Family medicine training institutions and organisations (such as WONCA Africa and the South African Academy of Family Physicians) have a critical role to play in supporting trainees and trainers towards developing local competencies which facilitate learning in the clinical workplace dominated by service delivery pressures.

## Introduction

This article reports on the findings of a workshop held at the joint 5th World Organisation of Family Doctors (WONCA) Africa and 20th National Family Practitioners Conference in Tshwane, South Africa, in August 2017. Postgraduate training for family medicine in Africa takes place in the clinical workplace at the bedside or next to the patient in the clinic, district hospital or regional hospital.^1,2^ Direct observation of the registrar by the supervisor with a learning conversation that evokes reflection and provides feedback is central to both training and assessment processes.^3^

Worldwide, workplace-based assessment (WBA) and learning has been recognised as the preferred way to train and assess postgraduate students.^4^ It is well known that learning and assessment are not dependent on any one method, but require a variety of educational strategies to deliver on the curriculum, which are best embedded in clinical practice as an authentic context.^5^ However, many challenges in the health service environment exist, which may impact on learning. In many instances, WBA boils down to a tick box exercise, where supervisors tick indicators or scores on a form, without giving any narrative feedback, and summative assessments may overshadow formative assessments. Even in high-income countries like the United Kingdom, many challenges exist in implementing a portfolio of learning to capture WBA.^6^ This has prompted the General Medical Council in the United Kingdom recently to suggest that one rather talks about the so-called supervised learning events (SLEs) instead of WBA.^7^

The World Organisation of Family Doctors and the South African Academy of Family Physicians (SAAFP) have established standards for the postgraduate training of family physicians.^8,9^ However, family medicine is a relatively new specialty in many African countries, which adds to the challenges around training and supervision in the context of large rural areas, massive health needs and minimal resources.^10^

The aim of the workshop was to understand how family medicine registrars (postgraduate trainees in family medicine) in Africa learn in the workplace. We particularly wanted to explore the interaction between the registrar and supervisor in the workplace, captured in a portfolio of learning, and in the African context. We sought a clearer understanding of what it means to be observed while conducting a consultation or performing a procedure, as well as understanding the local experience of giving or receiving feedback, and how various educational meetings are conducted.

## Participants and process

Thirty-five people participated in a 2-h workshop and included trainers and trainees from nine African countries, the United Kingdom, United States and Sweden (see Table 1). South Africa was represented by the universities of Cape Town, Limpopo, Pretoria, Sefako Makgatho, Stellenbosch, Walter Sisulu and Witwatersrand.

**TABLE 1:** Workshop participants.

Country	Number of participants
Botswana	2
Ethiopia	1
Ireland	1
Kenya	1
Lesotho	4
Malawi	3
Nigeria	2
South Africa	12
Sweden	1
Uganda	1
United Kingdom	2
United States (involved with Lesotho programme)	2
Zimbabwe	3

**Total**	**35**

We started with an introduction and then divided into buzz pairs (pairs were allowed to form spontaneously, regardless of the trainer or trainee status of the participants). In the buzz pairs, we explored the questions of how do I teach or learn, supervise or be supervised, and assess or be assessed. This was followed by an interactive focus group discussion on the reflections created by the buzz pair discussions (a guiding style was employed to facilitate this discussion). The group reflections were captured on a flip chart by a scribe. Common themes were identified. Clarification was sought and validated immediately with the workshop participants. A preliminary draft of this report was shared with the workshop participants after the conference.

## The major themes

Six major themes were identified and are discussed below.

### Context is critical

The physical place where trainees are based is a key factor in appropriate training for family medicine. There must be a balance between training in a district hospital and primary health care (PHC) settings. Participants spoke of accreditation of a ‘teaching campus’ in the district where training would be provided by a network of different facilities or locations. Rotation in specialist disciplines at referral hospitals tended to cause a sense of disconnect with the ethos of family medicine. In some programmes, registrars were based mostly in a tertiary setting, without on-site supervision by a family physician. Trainees then experienced a sense of dissonance, often missing the psychosocial aspects of patient care, as the specialist environment was mainly biomedical in nature. Without regular meetings with a family physician, it became a challenge to change the thinking of the trainee after his or her tertiary setting exposure.

The placement of registrars in the district was seen as strategic in terms of building the capacity of health teams. The risk, however, is that service provision overshadows learning, with a perception that the registrar is there to cover staff shortages. Indeed, registrars often provide supervision to mid-level health workers and medical assistants (similar to clinical officers or clinical associates).

Within the right context (district), the supervisor plays a key role in modelling to the registrar, for example, how to approach patients with challenging conditions in a comprehensive way, while making use of appropriate resources.

In some programmes, the initial training model started with international partners using a hub and spokes model, with training mainly occurring in ambulatory care settings (the spokes), linked with rotations in specialist departments (the hub of the regional hospital). A regular challenge is the geographic distances between the universities in the city and the district hospital in the periphery.

### Learning style of the registrar and (teaching style) of the supervisor

The success of the current educational emphasis on self-directed life-long learning was perceived to be heavily influenced by the previous learning background of the registrar. Some medical schools promoted more problem-based learning, with some emphasising self-directed learning, while others still adhered to mainly didactic teaching. This determined the registrar’s ability to drive their own learning. Furthermore, the previous learning and teaching exposure of the supervisor also became relevant, especially if their own training was influenced by a traditional specialty versus a family medicine approach. The importance of aligning learning styles and creating continuity between the undergraduate and postgraduate learning phases was recognised. Participants confirmed that the patient remains the best teacher. Registrars learnt best through seeing patients and then studying around them, as their learning needs were unmasked.

### Learning portfolio is utilised

The group explored the various components of a learning portfolio. In many programmes, a learning plan is drawn up per rotation, with objectives linked to the curriculum. Areas of existing competence and gaps in prior learning are identified and converted into learning objectives. Conversations with the supervisor help to develop a strategy to achieve the learning objectives. Participants were using tools like the mini-clinical evaluation exercise (CEX), logbook and direct observation of procedural skills (DOPS). These were often used at patient encounters and in meetings with their supervisor.

In terms of WBA, participants utilised a global assessment tool. Typically, there was one assessment at the end of each rotation, completed by both registrar and family physician supervisor, which was also used for shared reflection. There were also continuous assessments every 2 weeks within training modules, where the registrars presented a specific topic to the supervisor, with a short ‘quiz’ involving a number of questions on the topic. Some programmes had quarterly assessments.

One of the challenges experienced with the portfolio of learning, which is mostly paper-based at present, is implementation of the portfolio by the registrars during every day clinical practice, as well as training of supervisors (faculty development).

### Interactions between registrar and supervisor

We wanted to understand exactly how registrars and supervisors interacted in the workplace. Many programmes reported weekly contact sessions that included academic activities, for example a journal club. In some places, educational meetings were held weekly, with an agenda including a seminar, topic discussion or a DOPS. Participants spoke of an ‘educational prescription’ to ‘treat’ the identified learning needs of the registrar, which was a tool used daily, similarly to discussing the treatment for a patient, discussed with the consultant.

In some places where there was no immediate regular supervisor, participants made use of peer supervision from within the registrar’s context, by a senior registrar assigned by the main supervisor. Regarding being observed during patient consultations, participants mentioned that being videoed was an alternative to direct observation, as long as the patient had given permission. An 8–10-min video could then be viewed and assessed at a later stage by the supervisor. Many participants felt uncomfortable with being observed or had little experience and even less training in direct observation. Registrars should be comfortable during the observation, and supervisors needed to listen ‘educationally’. We explored the four simple rules for observation, as described by Holmboe^11^:

- **Correct positioning**: As the rater, try to avoid being in the line of sight of either the patient or trainee, especially when they are communicating. Use the principle of triangulation. However, during physical examinations be sure you can view the trainee’s techniques accurately.
- **Minimise external interruptions**: Let your staff know you will be with the trainee for 5–10 min, avoid taking routine calls or letting staff enter the room.
- **Avoid intrusions**: Don’t interject or interrupt if at all possible. Once you interject yourself into the trainee–patient interaction, the visit is permanently altered. However, there may be times at some point in the visit where you need to interject yourself in order to correct misinformation or risky management from the trainee.
- **Be prepared**: Know before you enter the room what your goals are for the observation session. For example, if a physical examination, have the trainee present the history first; then you will know what the key elements of the physical examination should be.

### Giving and receiving feedback

It was important to choose the timing of feedback. For example, when a registrar was post-call, this was not a good time for feedback. Feedback should not be given in front of the patient. However, the possibility of patients and other members of the health care team giving feedback in a multi-source format was seen as acceptable.

When giving feedback, it is important to allow the registrar to reflect first, and allow for time to reflect, with the supervisor being silent, or waiting. It is important to agree on what needs to be worked on. Feedback should be honest and specific. The relationship between the supervisor and the registrar may enhance or detract the potential for learning through feedback. The power gradient between supervisor and registrar may be a challenge, especially in many African cultures where seniority of age supersedes level of education.

A culture of feedback needs to be established in the institution or organisation. The same principles of feedback apply to undergraduates, postgraduates, junior and senior clinicians. Supervisors may also need to video their own consultations and reflect on their modelling ability. One participant stressed that ‘our health system needs more feedback’ and a blame-free learning culture. Giving and receiving feedback needs to be normalised, become part of everyday practice and not be seen as just for students. This requires building relationships, which are open for feedback.

When giving feedback, focus the conversation around the standard or norm (the issue at hand) which was not met (and not the person). This creates a triangle in which the issue becomes the focus and separate to the people receiving or providing feedback. This will help to avoid a confrontational style or tone. This approach may also be used in meetings or email conversations. Always aim to provide feedback which is constructive and not critical. It is important to give praise when things go well. Such compliments help to establish a culture of appreciation, build self-esteem and reinforce the correct attributes. And when things are not going well, feedback should be helpful and even rescuing. Open feedback between peers (registrar to registrar) was also encouraged.

The pros and cons of oral and written feedback were considered, as well as whether feedback should be given in person or via a ‘Skype call’. The value of face-to-face feedback allows for the registrar and supervisor to appreciate the non-verbal cues that contribute to communicating an unambiguous message. It is important to capture or document the feedback provided, especially for the registrar in difficulty. The registrar’s reflection (and supervisor’s reflection) on the feedback exchange should be captured in a journal or diary, as this will allow for follow-up conversations or reflections later on.

### The competence of the supervisor

The clinical competence of the clinical teacher should be good. The teacher or supervisor should be ‘clinically relevant’ (as opposed to ‘we never see him or her’). It is an accepted part of career progression that clinical supervisors may experience their roles evolving to less direct clinical contact. But it is important that the registrar feels that his or her supervisor ‘understands the clinical context’ and is able to ‘walk the walk and talk the talk’.^12^ This will enable the supervisor to provide meaningful feedback to a registrar who will value this feedback based on the credibility of the supervisor’s background.

## Reflection on workshop

The group appreciated the richness of the discussion and the value of having a variety of countries represented in the workshop. The group members expressed feeling encouraged and felt motivated to use ‘small moments, little bits, part of the mini-CEX’ during learning interactions in the workplace (teachable moments). This will allow the supervisors and registrars to be ‘more real’ in the workplace, as opposed to striving for the hard-to-reach perfect or ideal learning interactions. It will necessitate a more honest and pragmatic approach to harness these learning moments. Ongoing discussions are needed around the validity of continuous assessments in the workplace for national examinations, such as the Fellowship of the College of Family Physicians of South Africa (FCFP[SA]), and the contribution of the learning portfolio to exit examination results. Collaborative training projects, like Training the Clinical Trainers (TCT) project and ‘FamLEAP’ initiative, are trying to address the need for training of supervisors in South Africa and also now Malawi and other countries in Africa in basic workplace-based educational skills, such as formative assessment and giving feedback.^13^

## Conclusion

It was clear from this workshop discussion that the training of family physicians across Africa shares many common themes. However, there are also big differences among the various countries and even programmes within countries. The way forward would include exploring the local contextual enablers that influence the learning conversations between trainees and their supervisors. Family medicine training institutions and organisations (such as WONCA Africa and SAAFP) have a critical role to play in supporting trainees and trainers towards developing local competencies that facilitate learning in the clinical workplace dominated by service delivery pressures.
